# Differential Cardiac and Peripheral Vascular Low-Frequency Oscillation Responses to Voluntary Breath-Hold

**DOI:** 10.3390/life16071127

**Published:** 2026-07-07

**Authors:** Anton R. Kiselev, Olga M. Posnenkova

**Affiliations:** 1 Coordinating Center for Fundamental Research, National Medical Research Center for Therapy and Preventive Medicine, Moscow 101990, Russia; 2 Research Institute of Bio-digital Health Systems, Saratov State Medical University, Saratov 410012, Russia

**Keywords:** breath-hold, autonomic nervous system, heart rate variability, photoplethysmography, low-frequency oscillations, cardiovascular regulation, sympathetic activity

## Abstract

*Objective*. This study performed a comparative analysis of autonomic responses during voluntary breath-holds at inspiration and expiration by assessing low-frequency (LF) oscillations in heart rate (HR) and photoplethysmogram (PPG) signals. *Methods*. Eleven healthy volunteers underwent a modified head-up tilt test with breath-holds at inspiration and expiration in supine and standing positions. Electrocardiogram, finger PPG, and respiratory signals were recorded simultaneously. LF oscillation (0.04–0.15 Hz) amplitudes were assessed during spontaneous breathing and breath-hold phases. Relative changes in LF amplitudes (ΔLFA) were calculated for each signal, and the ΔLFA ratio (ΔLFA_PPG_/ΔLFA_HR_) was derived. *Results*. Cardiac and vascular signals showed divergent responses in LF oscillation amplitude during breath-holds. While PPG signals demonstrated a significant increase during expiration-holds (ΔLFA_PPG_ = +71.56%, *p* = 0.003), HR signals showed a non-significant overall decrease (ΔLFA_HR_ = −25.28%, *p* = 0.481). In this exploratory study (n = 11), comparative analysis showed that the vascular response (ΔLFA_PPG_) was significantly greater than the cardiac response (ΔLFA_HR_) during expiration-holds (*p* = 0.005) and across all stages (*p* = 0.012). However, expiration holds were systematically shorter than inspiration holds by approximately 24 s (median 32.1 s vs. 56.3 s). Because breath-hold duration was self-determined and not standardized, the observed differences between inspiration and expiration conditions may reflect either respiratory phase, cumulative apnea duration, or their interaction (our design cannot disentangle these effects). The ΔLFA ratio showed a negative median value overall (−0.25), indicating a complex and often inverse relationship between vascular and cardiac responses. However, due to the small sample size (n = 11), these results are strictly hypothesis-generating and cannot be generalized beyond the studied cohort. The study was powered only to detect large effect sizes (Cohen’s d > 1.2), and the wide bootstrap confidence intervals indicate substantial estimation uncertainty. Independent replication in larger, more diverse populations is essential before any clinical or physiological generalization can be made. *Conclusions*. This study documents opposing directional changes in cardiac and peripheral vascular LF oscillations during shorter expiration and longer inspiration breath-hold. Because respiratory phase and apnea duration are confounded in our design, we cannot determine whether these differential responses are phase-dependent, duration-dependent, or driven by both factors. The findings highlight the necessity of multi-signal analysis incorporating both ECG and PPG for a more comprehensive autonomic assessment of local and systemic autonomic influences and underscore that future studies must employ standardized breath-hold durations across both respiratory phases to isolate the specific contribution of respiratory phase. The ΔLFA ratio is explored here as a descriptive metric of the direction and magnitude of cardiac-vascular response differences, but its mathematical stability and physiological interpretation remain limited and require validation with direct sympathetic nerve recordings.

## 1. Introduction

Advancing the methodologies for investigating autonomic cardiovascular control continues to be a pertinent research objective. In this context, cardio-respiratory interactions play a pivotal role, involving complex mechanisms operating at multiple physiological levels. Respiratory sinus arrhythmia (RSA) is the most well-studied type of cardiorespiratory interaction. The magnitude of RSA depends on the depth and frequency of breathing [1], as well as the functional state of the organism [2]. According to most authors, the key mechanism underlying RSA is the baroreceptor reflex [3,4], which is mediated by the parasympathetic nervous system (vagal outflow). It is also known that a phenomenon of phase-frequency interaction, which can lead to synchronization and phase-locking, exists between oscillations in heart rate (HR) and the respiratory rhythm [5,6,7,8,9]. This creates a rationale for using respiration as a means to influence cardiac regulation. A related phenomenon is that of resonance breathing [10], where breathing slowed to six cycles per minute induces a significant amplification of low-frequency (LF) oscillations in HR, with a fundamental frequency around 0.1 Hz. An alternative approach to influencing cardiovascular regulation through respiration is voluntary breath-holding.

Research demonstrates that breath-holding induces complex cardiorespiratory adaptations involving multiple reflex mechanisms [11]. During breath-hold, contradictory heart rate responses occur due to varying sympatho-vagal balance between individuals, with modeling studies showing that heart rate typically increases during breath-holding in air [11]. Short-term cardiovascular adaptation to breath-hold, which is better studied in divers, involves rapid and complex physiological adjustments. During breath-hold, heart rate decreases within the initial 30 s, followed by a stable phase lasting 2–2.5 min, and potentially a third phase of continued bradycardia in experienced divers [12]. Blood pressure increases progressively throughout the breath-hold, rising 8 mmHg in the first phase and an additional 12 mmHg by the end [12]. Trained divers demonstrate faster onset of bradycardic responses and more rapid adjustments in systemic vascular resistance compared to non-divers [13]. Prolonged breath-holding causes marked left ventricular chamber enlargement, allowing stroke volume, cardiac output, and arterial pressure to be maintained despite reduced contractile function [14]. These cardiovascular changes are accompanied by alterations in cardiac output that affect pulmonary perfusion distribution and gas exchange patterns [15].

Breath-holding can be used as a model for understanding sleep apnea mechanisms and characteristics. Research demonstrates that the rate of oxygen saturation decline during breath-holding in wakefulness closely mirrors that observed during actual sleep apneas, with similar patterns regardless of whether obstructive or non-obstructive mechanisms are simulated [16]. Breath-holding maneuvers can serve as a clinical tool to assess elevated loop gain, a key phenotypic trait in obstructive sleep apnea, with reduced maximal breath-hold duration and larger ventilatory responses indicating hypersensitive ventilatory control [17]. Patients with obstructive sleep apnea exhibit significantly shorter breath-holding times compared to normal subjects and snorers, suggesting altered respiratory control mechanisms [18]. Computational modeling indicates that breath-holding induces complex cardiorespiratory adaptations involving reflex mechanisms that can help explain physiological responses during apneic events [11]. Given that the biomechanics of respiratory influences on cardiac regulation vary depending on the phase of the respiratory cycle [19], studying breath-holds separately at inspiration and expiration is of particular interest. The available literature lacks data on the short-term adaptation of the cardiovascular system to such breath-holds. This research could be valuable for a better understanding of the interactive mechanisms of cardiovascular adaptation.

LF oscillations (0.04–0.15 Hz) observed in HR [20,21], blood pressure [20,22], and peripheral blood flow, particularly in the photoplethysmogram (PPG) signal [23], attract researchers’ attention not only due to their direct role in the abovementioned resonance breathing but also as an important mechanism of autonomic regulation of systemic circulation. The physiological interpretation of LF oscillations remains actively debated. Many scientists associate these oscillations with baroreflex activity [24,25], while others link them to sympathetic modulation of peripheral vascular tone [25,26,27,28]. It is also thought that LF oscillations are involved in nonlinear cardiovascular interactions [29,30]. Therefore, studying the dynamics of LF oscillation properties during breath-hold, as a provocative test, is of direct interest.

This study aimed to compare the effects of inspiration and expiration breath-holds on HR variability and the features of LF oscillations in HR and PPG signals in healthy subjects. While both HR variability and PPG analysis are established techniques and breath-hold physiology is well described, no previous study has systematically compared the direction and magnitude of LF oscillation changes in cardiac (HR) and vascular (PPG) signals simultaneously during inspiration versus expiration breath-holds. Specifically, it remains unknown whether cardiac and peripheral vascular LF responses are uniformly coordinated or can dissociate depending on the phase of the respiratory cycle. Thus, the novelty of this study lies not in introducing a new measurement technique but in providing the first quantitative, within-subject comparison of differential cardiac-vascular LF responses to phase-specific breath-holds.

## 2. Materials and Methods

### 2.1. Subjects

The study involved 11 healthy subjects (6 males and 5 females) aged 22 ± 2 years.

Healthy volunteers were recruited from the student and staff populations of Saratov State Medical University through internal advertisements. Participants were eligible if they met the following criteria: (i) aged between 18 and 35 years; (ii) had a body mass index of 18.5–30 kg/m^2^; (iii) were in good general health as confirmed by medical history, physical examination, and laboratory tests; and (iv) provided written informed consent.

Volunteers were excluded for (i) significant past or present medical conditions; (ii) recent use of medication (within 4 weeks); (iii) substance abuse; (iv) positive screening tests for drugs, alcohol, or pregnancy; or (v) recent participation in another research study.

A total of 15 volunteers were initially screened. Four individuals were excluded: two did not meet the age criteria, one had a body mass index exceeding 30 kg/m^2^, and one was taking antihistamine medication within the exclusion window. The remaining 11 subjects were included in the final analysis.

Despite implementing body mass index limitations as an inclusion criterion, previous research has demonstrated that overweight and obesity do not affect indices of cardiac autonomic control [31].

### 2.2. Ethical Approval

The study protocol was approved by the Ethics Committee of the Saratov State Medical University (Protocol No. 1, 5 February 2019), and all experimental procedures were performed in accordance with the ethical standards laid down in the Declaration of Helsinki. All subjects were informed about the experimental procedures in detail and have signed standard consent forms.

### 2.3. Design of Study

We recorded electrocardiogram (ECG), PPG, and respiration simultaneously in the subjects during a modified passive head-up tilt test with breath-holds at inhalation and exhalation.

In our study, the experimental protocol consisted of four stages involving voluntary breath-hold: supine inspiration hold (Stage 1), supine expiration hold (Stage 2), standing inspiration hold (Stage 3), and standing expiration hold (Stage 4). The order of the four stages was fixed, proceeding sequentially from Stage 1 to Stage 4. This fixed order was chosen to minimize the cumulative physiological burden on participants, as breath-hold maneuvers can induce significant cardiorespiratory stress, and to maintain consistency with the standardized head-up tilt testing procedure, which typically begins in the supine position before proceeding to standing. The breath-hold was performed following an audio signal, which was delivered 30 s after the start of the test stage. The breath-hold duration was the maximum achievable for each individual subject. All participants received detailed instructions on this procedure prior to the test.

A recovery period of 5 min was provided between consecutive stages to ensure that heart rate, blood pressure, and respiratory patterns returned to baseline levels before the next maneuver.

All subjects were examined in the afternoon hours on an empty stomach. The room temperature was within a comfortable range (22–23 °C).

### 2.4. Signal Recording

We analyzed 44 records of simultaneous ECG, PPG from the left index finger, and respiration obtained from 11 subjects during all four stages of the head-up tilt test. Respiration was recorded using a flow sensor (nasal cannula) to capture the respiratory waveform. The length of each recording was defined by the maximum breath-hold duration achievable by each individual subject. All signals were sampled at 250 sps and digitized at 14 bits.

All experimental signals were recorded using an electroencephalograph analyzer EEGA-21/26-Encephalan-131-03 (Medicom MTD LLC, Taganrog, Russia). Only ECG, PPG, and respiration recordings without artifacts, extrasystoles, and pronounced trends were left for further analysis.

### 2.5. Signal Preprocessing

For HR variability analysis, R peaks were detected from the ECG signal using a robust algorithm combining methods from the BioSPPy 2.2.2 library and adaptive threshold-based peak detection. The sequence of RR intervals (time between consecutive R peaks) was extracted and filtered to remove physiologically unrealistic values (RR < 0.3 s or RR > 2.0 s).

To transform the non-equidistant RR interval series into an equidistant time series suitable for analysis, we applied linear interpolation of both RR intervals and instantaneous HR values (60/RR) onto a regular time grid with a sampling frequency of 10 Hz. This approach preserves the temporal characteristics of HR variability while creating uniformly sampled signals.

Respiration and PPG signals were synchronized with the ECG-derived data by truncating to the common time range defined by the first and last detected R peaks, followed by linear resampling to the same 10 Hz frequency. All signals were thus aligned to a unified timeline for subsequent analysis. In all 44 recordings (11 subjects × 4 stages), ECG, PPG, and respiratory signals were simultaneously acquired and available for analysis without loss or corruption.

No additional filtering or preprocessing was applied to the respiration and PPG signals beyond the described synchronization and resampling procedure.

### 2.6. Protocol Execution and Timing Quantification

The respiratory signal for each recording was analyzed to objectively quantify subject compliance with the breath-hold protocol and the temporal structure of the maneuver.

The respiratory signal was analyzed to identify breath-hold and spontaneous breathing phases. Breath cycles were identified by detecting peaks and troughs in the bandpass-filtered (0.1–0.5 Hz) respiratory waveform. Breath-hold phases were automatically detected when the interval between consecutive respiratory events exceeded a dynamic threshold based on the median respiratory cycle duration (typically >3× median duration or ≥5 s). Spontaneous breathing phases were defined as periods with regular respiratory activity lasting ≥10 s. All identified phases were validated visually using generated respiration and heart rate plots.

Each recording was subsequently segmented into two distinct phases: a spontaneous breathing phase and a breath-hold phase (further categorized into inspiration-hold and expiration-hold).

The resulting phase segmentation was used to calculate phase duration ratios and extract phase-specific metrics of HR variability, RSA and LF oscillations in HR and PPG.

### 2.7. Signal Processing

Analysis of signals revealed a mean recording length of 77.8 ± 16.7 s per stage.

This short-term design, capturing the immediate autonomic transition into and out of the breath-hold state, precluded the use of classical power spectral density (PSD) analysis. PSD estimation requires stationary signals of sufficient duration (typically 2–5 min for reliable LF power estimates [32]), and our segments (28.2–61.5 s) fall well below this threshold. Attempting PSD analysis on such short, non-stationary segments would yield statistically unreliable estimates due to insufficient degrees of freedom, frequency smearing, and violation of stationarity assumptions. Consequently, the analysis was focused on robust time-domain metrics of HR variability and the assessment of LF oscillation amplitudes in HR and PPG signals via digital filtering and time-domain feature extraction, which have been validated for the analysis of ultra-brief (≤60 s), non-stationary physiological signals [33,34,35]. This approach quantifies the magnitude of LF oscillations (standard deviation of the bandpass-filtered signal) without requiring stationarity or long recording durations.

This short-term design, capturing the immediate autonomic transition into and out of the breath-hold state, precluded the use of classical spectral analysis for absolute power estimates. Consequently, the analysis was focused on robust time-domain metrics of HR variability and the assessment of LF oscillation amplitudes in HR and PPG signals via digital filtering and time-domain feature extraction, which are validated for the analysis of ultra-brief, non-stationary physiological signals [33,34,35].

### 2.8. Time-Domain HR Variability Analysis

Standard deviation of RR intervals (SDNN) and the root mean square of successive differences (RMSSD) were calculated as measures of overall and parasympathetic-mediated HR variability, respectively [32]. The analysis was performed separately for spontaneous breathing and breath-hold phases. Poincaré plot parameters (SD1, SD2) were also calculated, where SD1 represents short-term variability and SD2 represents long-term variability. Sample entropy was computed as a measure of signal complexity.

### 2.9. Respiratory Sinus Arrhythmia Quantification

The amplitude of RSA was calculated using the peak-to-trough (PtT) method, defined as the difference between the maximum and minimum RR interval within a single respiratory cycle during the spontaneous breathing phase. The analysis was performed on the RR interval series without outlier filtering to preserve respiratory-related variations, as conventional outlier removal methods (e.g., threshold-based rejection of physiologically implausible RR intervals) may inadvertently eliminate genuine respiratory-related fluctuations, particularly those occurring at the extremes of the respiratory cycle. To ensure that our findings were not biased by potential outliers, we conducted a sensitivity analysis by visually inspecting all RR interval series for ectopic beats and artifacts prior to RSA calculation. No ectopic beats or significant artifacts were detected in the segments used for RSA analysis. Additionally, we recomputed RSA amplitudes after applying a mild outlier correction (removal of RR intervals deviating by >30% from the preceding interval) and confirmed that the median RSA values remained virtually unchanged, with the median absolute difference across subjects being less than 3%. This confirms that the uncorrected approach did not introduce systematic bias. For each subject, the median RSA amplitude across respiratory cycles was used.

### 2.10. Analysis of LF Oscillations

A comparative analysis of LF oscillations (0.04–0.15 Hz) was performed using simultaneously recorded HR and PPG signals. For each subject and stage, the signals were first segmented into SB and BH phases based on the synchronized respiratory signal. Each segment was then processed using an identical methodology.

To isolate the LF oscillatory component, both signals were filtered with an identical zero-phase bandpass Butterworth filter (0.04–0.15 Hz). Prior to filtering, the RR chain was converted to an HR signal (beats/min) using the formula HR = 60,000/RR [32], and both signals were resampled to a uniform 4 Hz grid using linear interpolation to ensure consistent frequency analysis. This resampling step was necessary to ensure that the two signals had identical temporal alignment and sampling rates for subsequent filtering and amplitude analysis. A sampling rate of 4 Hz was selected as it exceeds the Nyquist frequency for the LF band of interest (0.15 Hz), thereby preventing aliasing while avoiding oversampling that could introduce unnecessary computational complexity. Importantly, the analysis focuses on the amplitude of LF oscillations (quantified as the standard deviation of the filtered signal) rather than on spectral power estimates; the chosen interpolation approach has been validated for such amplitude-based assessments of ultra-brief physiological signals [33,34,35].

To account for interindividual differences in PPG signal amplitude that arise from technical (sensor placement, contact pressure) and physiological (skin perfusion, tissue optical properties) factors, we applied a normalization procedure prior to calculating LF oscillation amplitudes. For each subject and stage, the raw PPG signal was normalized by dividing by its mean value during the spontaneous breathing phase of that stage. This approach yields a dimensionless signal expressed as a percentage of the baseline amplitude, thereby removing subject-specific gain factors while preserving the temporal oscillatory structure. All subsequent LFA calculations for PPG signals were performed on these normalized signals. This normalization strategy is conceptually similar to that used in previous PPG variability studies (see, e.g., ref. [26]) and enables meaningful interindividual comparisons of relative oscillation amplitude. Importantly, this normalization does not affect the calculation of ΔLFA (relative change from breathing to breath-hold), which was already a within-subject measure, but it improves the interpretability of absolute LFA values across subjects.

For each resulting filtered segment, the following parameters were calculated. The amplitude of the LF oscillations (LFA) was quantified as the standard deviation of the bandpass-filtered signal, which provides a robust measure of oscillation power in the time domain.

The LFA of the PPG signal was expressed in arbitrary units (a.u.) of its raw amplitude to account for individual differences in signal gain and peripheral blood flow.

The relative change in LFA (ΔLFA) was calculated for each signal (ΔLFA_PPG_ and ΔLFA_HR_, respectively), subject, and stage as the difference (in percentage) between the LFA under the breath-hold phase (LFA_hold_) and spontaneous breathing phase (LFA_breathe_): ΔLFA = [(LFA_hold_ − LFA_breathe_)/LFA_breathe_] × 100%.

The relationship between LF oscillations in HR and PPG was evaluated by calculating the ratio of their ΔLFA: ΔLFA ratio = ΔLFA_PPG_/ΔLFA_HR_.

### 2.11. Statistical Analysis

Due to the non-normal distribution of data (assessed by the Shapiro–Wilk test), non-parametric statistics were applied. The Wilcoxon signed-rank test was applied for within-stage comparisons and the Mann–Whitney U test for between-group comparisons. The Friedman test was used to assess the overall effect of the four-stage protocol on studied parameters. A *p*-value of less than 0.05 was considered statistically significant. Data processing and statistical analysis were performed using custom scripts and algorithms developed in Python 3.11.

No formal a priori power calculation was performed. Given the sample size of 11 participants, the study was adequately powered only to detect large effect sizes (Cohen’s d > 1.2) and is therefore exploratory in nature. Smaller but potentially meaningful differences may remain undetected, and all *p*-values should be interpreted conservatively; absence of statistical significance (*p* > 0.05) does not imply equivalence. Consequently, the obtained results cannot be used for broad generalization to the general population or clinical populations. The primary purpose of this study is strictly hypothesis generation rather than definitive inference. Nevertheless, the robustness of the locally detected effects is supported by (i) the use of a within-subject design, which controls for inter-individual baseline variability; (ii) simultaneous recording of cardiac and vascular signals, ensuring temporal alignment and internal consistency; (iii) the large effect sizes observed for significant comparisons (e.g., rank-biserial correlation r = 0.933 for ΔLFA_HR_ decrease during Stage 2; Cliff’s delta = 0.975 for the difference between ΔLFA_PPG_ and ΔLFA_HR_ during Stage 2); and (iv) the consistency of the vascular-dominant response pattern across both supine and standing expiration holds. However, these factors support internal validity only and do not extend to external validity or generalizability. Bootstrap 95% confidence intervals for medians were calculated using the percentile method with 5000 resamples. These intervals are wide (see Section 3. ) and reflect substantial estimation uncertainty due to the small sample size. They should not be interpreted as precise estimates of population parameters.

For PPG signals, the normalization procedure described in Section 2.10 was applied to all subjects. To verify the effectiveness of this normalization in reducing interindividual variability, we calculated the coefficient of variation (CV) for LFA_PPG_ values during spontaneous breathing before and after normalization. The CV decreased from 62.4% to 34.1% after normalization, indicating successful reduction in between-subject variance attributable to technical gain factors.

The ΔLFA ratio is mathematically problematic when ΔLFA_HR_ values are near zero or change sign. No formal stability analysis was performed. Therefore, this ratio should be considered an exploratory descriptive metric, not a validated physiological index. All ratio-derived interpretations are provisional.

For significant Wilcoxon signed-rank test results (within-stage comparison of ΔLFA to zero), effect sizes were calculated as rank-biserial correlation r = 1 − (2W)/(n·m), where W is the Wilcoxon statistic and n·m is the product of sample sizes (interpretation: r ≥ 0.5 large, ≥0.3 medium, ≥0.1 small). For between-signal comparisons (Mann–Whitney U test), we calculated Cliff’s delta with the same threshold magnitudes.

Bootstrap 95% confidence intervals for medians were calculated using the percentile method with 5000 resamples to quantify estimation uncertainty, given the small sample size.

## 3. Results

### 3.1. Temporal Structure of the Protocol

**Figure 1:**
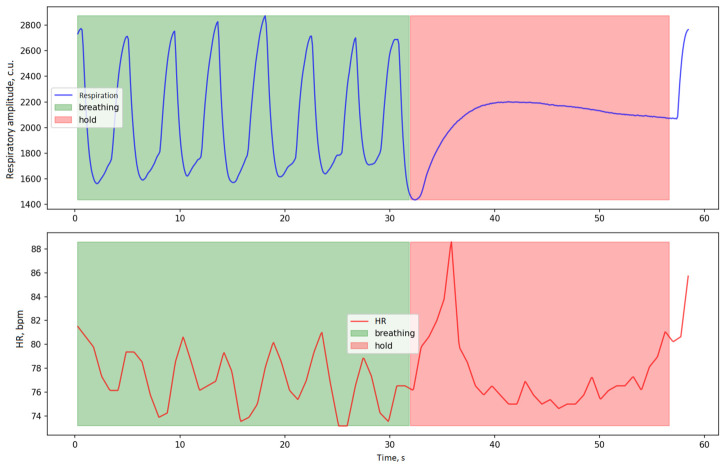
Representative recording from one subject during Stage 4 (standing expiration hold), including the respiratory signal and heart rate signal. The spontaneous breathing phase is highlighted in green, and the expiration breath-hold phase is highlighted in red. This example illustrates the clear segmentation of phases.

Analysis of the respiratory signals confirmed high subject compliance with the instructed protocol. The breath-hold phases were clearly identifiable in all 44 recordings (e.g., Figure 1).

### 3.2. Breath-Hold Duration

The duration of voluntary breath-hold was significantly influenced by the phase of the respiratory cycle at which it was initiated (Table 1). Breath-holds at the end of inspiration (Stages 1 and 3) were substantially longer than breath-holds at the end of expiration (Stages 2 and 4). This difference was consistent across both supine and standing positions (Table 1).

It is worth noting that the stages were performed in a fixed order (supine inspiration hold, supine expiration hold, standing inspiration hold, standing expiration hold). Consequently, expiration holds were always performed after inspiration holds in the same posture. While this design allowed for consistent procedural standardization, we cannot entirely exclude the possibility that order effects, such as habituation or mild fatigue, may have contributed to the observed differences in breath-hold duration. However, the consistent pattern across both supine and standing positions, with inspiration holds being longer regardless of whether they appeared as the first or third stage, suggests that the respiratory phase itself is the primary determinant.

### 3.3. Time-Domain HR Variability and RSA Parameters

Both SDNN and RMSSD during spontaneous breathing were significantly affected by the experimental stage (Table 2). This pattern was similarly reflected in the Poincaré plot parameters, with both SD1 (representing short-term variability) and SD2 (representing long-term variability) during spontaneous breathing showing significant stage-dependent changes (*p* = 0.007 and *p* = 0.005, respectively). The highest values of these parameters during spontaneous breathing were observed at Stage 1, while the lowest values were observed at Stage 4. Notably, the reduction in SD1 from Stage 1 to Stage 4 was more pronounced than that of SD2. The amplitude of respiratory sinus arrhythmia (RSA PtT) during spontaneous breathing phases was numerically higher in the supine position (Stages 1 and 2) compared to the standing position (Stages 3 and 4), though this difference was not statistically significant (*p* = 0.126). Sample entropy during breath-hold phases showed no significant overall differences (*p* = 0.284).

### 3.4. Comparative Dynamics of LF Oscillations in HR and PPG Signals

LF oscillations were present in all recordings. Their characteristics were significantly modulated by the experimental conditions.

The amplitude of LF oscillations in HR (LFA in HR) showed significant variations across stages during spontaneous breathing (*p* = 0.024), but not during breath-hold phases (*p* = 0.122). The highest LFA in HR during spontaneous breathing was observed at Stage 3, while the highest median LFA in HR under breath-hold was observed at Stage 3.

In contrast, LF oscillations in PPG (LFA in PPG) did not show significant stage-dependent differences during spontaneous breathing (*p* = 0.689) or breath-hold phases (*p* = 0.060).

The response patterns differed between signals: while LFA in HR generally decreased from breathing to hold phases in supine positions (Stages 1–2), it showed more variable changes in standing positions (Stages 3–4). LFA in PPG demonstrated heterogeneous responses with notable interindividual variability.

The relative change in LF oscillation amplitude (ΔLFA) from spontaneous breathing to breath-hold showed distinct patterns between HR and PPG signals (Table 3).

For HR signals, ΔLFA_HR_ was predominantly negative across conditions, indicating a general decrease in LF oscillation amplitude during breath-hold. The most pronounced decrease was observed during supine inspiration hold (Stage 1: −52.42%, *p* = 0.383). A significant decrease was detected during supine expiration hold (Stage 2: −32.38%, *p* = 0.020). The rank-biserial correlation for this decrease was r = 0.933, indicating a large effect, while no other within-stage changes were significant for ΔLFA_HR_ (Table 3).

For PPG signals, ΔLFA_PPG_ showed more heterogeneous responses. A significant increase was observed during supine expiration hold (Stage 2: +71.56%, *p* = 0.004) and when combining all shorter expiration holds (+71.56%, *p* = 0.003). The overall ΔLFA_PPG_ across all stages was also significantly positive (+49.75%, *p* = 0.003) (Table 3).

**Figure 2:**
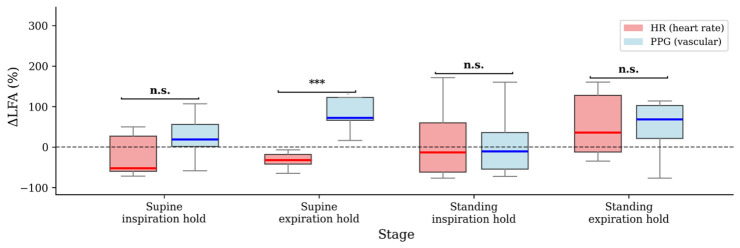
Relative change in low-frequency oscillation amplitude (ΔLFA) from spontaneous breathing to breath-hold in heart rate (HR) and photoplethysmogram (PPG) signals across experimental stages. Box plots show median (horizontal line), interquartile range (box), and range (whiskers). Positive values indicate increased LF oscillation amplitude during breath-hold. Significant within-stage differences between HR and PPG are indicated ( *** *p* < 0.001). Stage 1: supine inspiration hold; Stage 2: supine expiration hold; Stage 3: standing inspiration hold; Stage 4: standing expiration hold.

Comparative analysis revealed significant differences between cardiac and vascular responses. These differences are illustrated in Figure 2, which shows the divergent ΔLFA responses between HR and PPG signals, particularly during shorter expiration-hold maneuvers. During supine expiration hold (Stage 2), ΔLFA_PPG_ was significantly greater than ΔLFA_HR_ (*p* = 0.001). The effect size for this difference was large (Cliff’s delta = 0.975). This differential response persisted when grouping all expiration holds (*p* = 0.005) and for all stages combined (*p* = 0.012) (Table 3).

The bootstrap 95% confidence intervals for ΔLFA medians are wide across all stages (e.g., Stage 2: ΔLFA_HR_ [−58.7, −7.3]%; ΔLFA_PPG_ [25.4, 388.1]%; see Table 3 footnotes), reflecting substantial inter-individual variability and the small sample size. These wide intervals preclude precise estimation of population parameters and underscore that the reported median values are sample-dependent point estimates only. While the direction of the vascular-dominant response is consistent, the magnitude of the effect in the broader population remains highly uncertain.

The ΔLFA ratio values in Table 3 show a mix of positive and negative median values (0.35, −2.21, −0.80, 0.73 across stages), with an overall median of −0.25 for all stages combined (Table 3). This indicates complex, often inverse relationships between cardiac and vascular responses rather than consistent proportional amplification.

To explore whether the observed ΔLFA values were confounded by breath-hold duration, we performed Spearman correlation analysis between BH duration and ΔLFA_PPG_ and ΔLFA_HR_ separately for inspiration and expiration holds. No significant correlations were detected. However, this analysis is limited for three reasons: (i) the sample size (n = 11) provides insufficient statistical power to detect moderate correlations; (ii) the duration range within each phase is narrow (inspiration holds: IQR 48.4–65.8 s; expiration holds: IQR 25.4–39.0 s), compressing the independent variable and reducing correlation sensitivity; and (iii) the analysis treats phase as a binary variable while duration is continuous, yet the two are almost perfectly collinear in our data (inspiration holds are always longer than expiration holds). Consequently, these null findings cannot be interpreted as evidence that duration is not a confounding factor. Rather, they reflect the main limitation that our design conflates phase and duration, rendering any attempt to isolate their independent effects statistically invalid.

### 3.5. Discussion

This study provides a strictly exploratory, hypothesis-generating comparative assessment of autonomic responses to voluntary breath-hold by simultaneously analyzing LF oscillations in cardiac (HR) and vascular (PPG) signals in a small cohort (n = 11). The results presented here cannot be generalized beyond the studied sample and should not be interpreted as representative of the general population or clinical populations. To the best of our knowledge, this is the first demonstration that during breath-hold, LF oscillations in HR and PPG can change in opposite directions depending on the phase of the respiratory cycle. While LF analysis and breath-hold physiology are individually well established, the differential, phase-dependent dissociation between cardiac and vascular LF responses has not been previously quantified. Thus, the primary novelty of this study is phenomenological: it documents a locally robust divergence in cardiovascular oscillatory patterns between the cardiac and peripheral vascular signals. Any interpretation of this divergence in terms of ‘autonomic outflows’ or specific neural mechanisms remains speculative, as LF oscillations are indirect, baroreflex-mediated measures rather than direct indices of autonomic activity.

The results demonstrate a complex autonomic response. The key finding of our study is the demonstration of distinct, rather than uniformly coordinated, patterns of LF oscillation in cardiac and vascular signals during voluntary breath-holds of differing durations. While these findings are internally consistent and supported by large effect sizes for significant comparisons (e.g., Cliff’s delta = 0.975 for Stage 2), they are derived from a small sample and cannot be broadly generalized. We found condition-dependent differences between cardiac and vascular responses, with a particularly strong contrast during the shorter expiration-hold maneuvers. The most striking observation was the opposing directional changes in LF oscillation amplitude: while PPG signals generally showed positive ΔLFA values (indicating increased vasomotor activity), HR signals demonstrated predominantly negative ΔLFA values (suggesting decreased cardiac autonomic modulation) during breath-hold. This inverse relationship is reflected in the negative median ΔLFA ratio value across all conditions. The terms ‘vasomotor activity’ and ‘cardiac autonomic modulation’ used here are functional descriptors, not direct neural measurements. However, the wide bootstrap confidence intervals and interquartile ranges indicate substantial uncertainty in the magnitude of these effects. These directional patterns should be viewed as preliminary hypotheses requiring independent replication in larger cohorts.

Within the limits of this small-sample study, this differential response suggests (but does not conclusively disprove) the concept of uniformly coordinated autonomic activation. Independent replication in larger cohorts is required. The vascular-dominant pattern observed during the shorter expiration-hold conditions (significant ΔLFA_PPG_ increase of +71.56%) is consistent with enhanced peripheral vasomotor activity. However, the PPG signal alone cannot distinguish between sympathetic, myogenic, or passive hemodynamic contributions.

**Figure 3:**
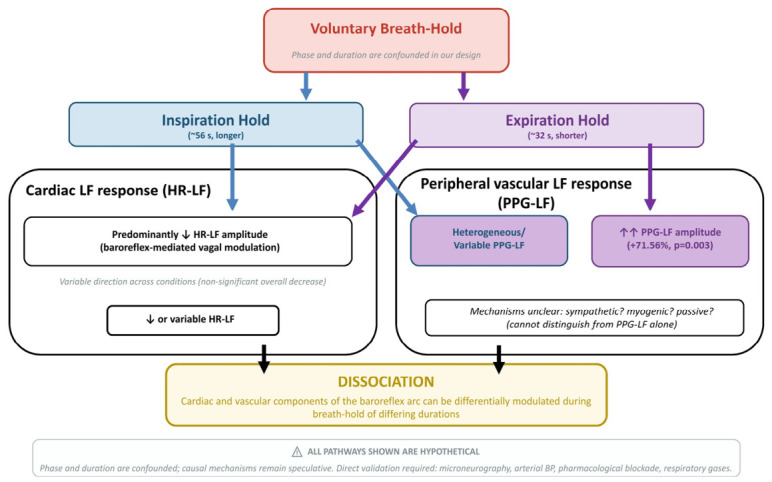
Conceptual model of differential cardiac and peripheral vascular low-frequency (LF) oscillation responses during voluntary breath-hold at different respiratory phases. Top: The two experimental conditions differed in both respiratory phase and self-determined breath-hold duration (confounded in our design). Left panel (Cardiac LF response, HR-LF): Both inspiration and expiration holds were associated with predominantly decreased HR-LF amplitude, hypothesized to reflect baroreflex-mediated vagal modulation rather than direct sympathetic cardiac tone. The direction was variable across conditions and non-significant overall. Right panel (Peripheral vascular LF response, PPG-LF): The vascular response diverged by condition: inspiration holds produced heterogeneous/variable PPG-LF changes, whereas shorter expiration holds produced a robust increase in PPG-LF amplitude (+71.56%). The underlying mechanisms (sympathetic vasoconstriction, local myogenic autoregulation, or passive hemodynamic redistribution) cannot be distinguished from PPG-LF amplitude alone. Bottom: The divergent directional changes suggest that cardiac and vascular components of the baroreflex arc can be differentially modulated during breath-hold of differing durations. All pathways shown are hypothetical and require validation through direct measurements (e.g., muscle sympathetic nerve activity, arterial blood pressure, pharmacological blockade, respiratory gas analysis). The figure is intended as a conceptual aid for hypothesis generation, not as established physiology.

The most plausible explanation for this phenomenon is the differential contribution of autonomic efferent pathways to the overall compensatory response (Figure 3). Figure 3 presents a conceptual model of the proposed pathways; all mechanisms depicted remain hypothetical and require direct experimental validation. The interpretation of LF oscillations in HR variability as a marker of sympathetic cardiac tone [36,37] has been contested. Evidence suggests it may reflect baroreflex-mediated modulation of parasympathetic outflow rather than direct sympathetic activation [38,39,40,41]. However, Cooley et al. [42] suggested a central autonomic origin of LF oscillations in HR beyond baroreflex mechanisms. In contrast, several investigations indicate that LF oscillations in the PPG signal may serve as a viable tool for evaluating neurogenic (sympathetic) control of peripheral vascular tone [43,44,45], as well as blood pressure regulation mechanisms [23]. Contrary evidence suggests that autoregulatory processes in microcirculation may decouple LF oscillations from central hemodynamic control [46,47,48,49]. If interpreted cautiously in autonomic terms, the data are compatible with a model in which the primary compensatory response to the hemodynamic challenge of breath-hold involves enhanced peripheral vascular tone (as reflected in increased PPG-LF amplitude), while cardiac LF modulation (as reflected in HR-LF amplitude) shows a different trajectory. However, this is one of several possible interpretations. We present this vascular-dominant pattern as a descriptive observation, not as evidence of sympathetically mediated vasoconstriction.

This vascular-dominant response was particularly evident during the shorter expiration-hold conditions, which were also the most potent provocateur in our protocol. The profound changes in intrathoracic pressure during prolonged expiratory apnea drastically reduce venous return and cardiac output, which likely necessitates a stronger vasoconstrictor response to maintain arterial pressure [50,51,52]. Our data quantify the enhanced vascular LF oscillatory response under these conditions, though whether this represents active vasoconstriction, passive hemodynamic redistribution, or baroreflex-mediated oscillatory behavior cannot be determined from LF amplitude alone. Similar physiological processes were previously studied in divers during breath-hold. Studies demonstrate that cardiac output decreases substantially during breath-hold dives [53,54]. This decreased cardiac output results from both bradycardia, with heart rates falling to 20–30 beats/min, and reduced stroke volume [53,54]. The cardiovascular system compensates through peripheral vasoconstriction, leading to dramatic increases in arterial blood pressure reaching values as high as 280/200 mmHg. These hemodynamic changes are accompanied by anaerobic metabolism and increased blood lactate concentrations [53].

Our finding highlights the critical value of incorporating vascular signals like PPG into autonomic assessment protocols. Relying solely on HRV analysis may underestimate the full magnitude of the baroreflex response, as it misses the dominant vascular component. The PPG signal provides a unique and non-invasive window into peripheral vasomotor control, which is often interpreted as reflecting sympathetic influences on the local vasculature when assessed under conditions of controlled central hemodynamics, though direct validation (e.g., via microneurography) is lacking in our study. However, it is crucial to acknowledge that the observed LF oscillations in PPG could also arise from local autoregulatory processes or passive pressure-flow relationships [46,47,48,49]. The ΔLFA ratio, showing a negative median in our study, is explored as a descriptive metric of directional balance. However, its mathematical stability is sensitive to ΔLFA_HR_ values near zero, and no formal validation was performed. Thus, the ratio should be interpreted with caution and used only for hypothesis generation. The condition-specific patterns (e.g., strong negative ratio during supine expiration hold: −2.21) suggest this metric may be useful for characterizing different autonomic response profiles. The within-recording comparison of conditions provides evidence that voluntary apnea modulates cardiovascular oscillatory patterns. The differential effects observed between shorter expiration-hold and longer inspiration-hold conditions may reflect baroreflex engagement, though blood pressure was not directly measured and baroreflex sensitivity could not be calculated. Any attribution to autonomic outflows is inferential. The stark contrast between shorter expiration-hold and longer inspiration-hold conditions underscores the potential importance of the interplay between respiratory phase and apnea duration in designing and interpreting autonomic provocation tests. However, because these factors are confounded in our design, we cannot determine which variable is primarily responsible for the observed differences.

In summary, this study does not claim to validate a new clinical biomarker (ΔLFA ratio) or to resolve the physiological origin of LF oscillations. The conceptual model presented in Figure 3 is offered solely as a visual aid for the hypothetical interpretation discussed above; it does not represent established mechanisms. It provides, for the first time, a quantitative description that during shorter expiration breath-hold conditions, vascular LF amplitude robustly increases while cardiac LF amplitude does not and that the two can dissociate in direction. Because phase and duration are confounded in our design, this observation describes the experimental condition rather than establishing a phase-specific effect.

*Interpretational caution*. The interpretation of LF oscillations in both HR and PPG signals remains a subject of ongoing debate. Moreover, the absence of respiratory gas measurements and tidal volume quantification precludes attribution of the observed responses to specific chemoreflex or mechanical pathways. While some studies associate LF power in PPG with sympathetic vasomotor control, others emphasize contributions from myogenic, endothelial, and respiratory mechanisms. Similarly, LF oscillations in HR variability are not a direct measure of cardiac sympathetic tone but rather reflect baroreflex-mediated modulation, predominantly via parasympathetic outflow. Therefore, the terms «sympathetic» or «autonomic» used in this study should be understood as functional correlates, not direct neural measurements. Alternative interpretations of the observed differential LF responses are possible, and the present findings do not resolve these controversies but add descriptive evidence under breath-hold conditions.

A critical caveat to all comparisons between inspiration and expiration holds is the systematic difference in breath-hold duration. Inspiration holds were approximately 24 s longer than expiration holds (median 56.3 s vs. 32.1 s; Table 1). This confounding is not incidental but structural: because participants self-determined their maximum breath-hold duration, and because end-inspiratory apnea is physiologically easier to sustain than end-expiratory apnea, the two phases produced non-overlapping duration distributions. Consequently, any observed difference between inspiration and expiration holds may reflect respiratory phase, apnea duration, or both, and our design provides no statistical basis to isolate their independent contributions. We critically consider three competing explanations for our findings. First, a phase-dependent mechanism: end-expiration is associated with reduced lung volume, increased intrathoracic pressure, and impaired venous return compared to end-inspiration. These mechanical differences could independently engage baroreflex and chemoreflex pathways, producing the observed vascular-dominant response during expiration holds. The consistency of the vascular-dominant pattern across both supine and standing expiration holds (Stages 2 and 4) lends some support to this interpretation. Second, a duration-dependent mechanism: longer apneas (inspiration holds) allow greater accumulation of hypoxia and hypercapnia, which may progressively alter autonomic outflows. The vascular response during shorter expiration holds may represent an earlier phase of autonomic adaptation, whereas longer inspiration holds may transition into a different regulatory regime. Third, an interaction mechanism: phase and duration may act synergistically, with the mechanical conditions at end-expiration accelerating the autonomic response that would otherwise require longer apnea times to manifest. Our exploratory Spearman correlation analysis between breath-hold duration and ΔLFA values (within each phase separately) yielded no significant associations, but this analysis is uninformative due to narrow duration ranges and insufficient power (see Section 3. Results). Therefore, we cannot exclude the possibility that the observed ‘phase-dependent’ effects are, in fact, duration-dependent effects masquerading as phase effects. Until future studies employ standardized breath-hold durations (e.g., fixed 30 s apneas) or a crossover design with matched apnea times, claims of phase-specificity remain speculative.

Thus, the primary contribution of this study is not to resolve the physiological origin of LF oscillations but to document a robust, condition-dependent differential response between cardiac and vascular signals. The findings stand regardless of whether the LF oscillations in the PPG signal is interpreted as sympathetic, myogenic, or baroreflex-mediated: the dissociation between HR and PPG responses during breath-holds is evident and warrants further mechanistic investigation.

We acknowledge that the individual methods used (HRV analysis, PPG, breath-hold maneuvers) are not novel. Likewise, the ΔLFA ratio is an exploratory descriptive metric that requires validation. However, the key finding, that voluntary breath-hold at expiration produces a significant increase in vascular LF amplitude while cardiac LF amplitude simultaneously decreases, has not been reported previously. This dissociation suggests that the cardiac and vascular components of the baroreflex arc can be independently modulated, a phenomenon that may have implications for understanding autonomic dysregulation in conditions such as orthostatic intolerance or heart failure.

## 4. Conclusions

In this small-scale hypothesis-generating study, voluntary breath-hold elicits condition-dependent and differentially coordinated responses in cardiac and vascular LF oscillations. The relationship between these systems is not uniformly correlated, with significant differences observed primarily during the shorter expiration-hold maneuvers. However, due to the limited sample size, differences in breath-hold duration between inspiration and expiration phases, and wide interindividual variability, these findings must be interpreted as preliminary hypotheses only. Independent replication in larger, more diverse populations is required before the ΔLFA ratio can be considered a sensitive or reliable indicator of vascular-cardiac response dissociation.

The response is qualitatively distinct, with the peripheral vascular bed typically showing increased LF oscillation amplitude during breath-hold, while cardiac responses are more variable and often show decreased modulation. This differential pattern underscores the complex nature of autonomic compensation and highlights the necessity of multi-signal analysis (ECG with PPG) for a comprehensive assessment of the interplay between systemic cardiac and local peripheral vascular responses, moving beyond the traditional cardiocentric view provided by HR variability alone. While the local vascular signal provides valuable insight into peripheral dynamics, its generalization to systemic vascular resistance requires caution.

The main contribution of this study is the phenomenological description of a previously unreported dissociation between cardiac and vascular LF oscillations during breath-hold. During the shorter expiration-hold conditions (median 32.1 s), vascular LF amplitude increased (+71.56%), while cardiac LF amplitude showed a non-significant decrease (−25.28%). During the longer inspiration-hold conditions (median 56.3 s), the patterns differed. Because phase and duration are confounded, these observations describe the experimental conditions rather than establishing causally distinct physiological states. This finding would not be captured by HRV analysis alone. The exploratory ΔLFA ratio is presented only as a descriptive index of this dissociation, not as a validated clinical metric. Independent replication with larger samples and direct sympathetic nerve recordings and concurrent measurements of blood pressure, oxygen saturation, and CO_2_ is required to confirm the underlying mechanisms.

The ΔLFA ratio is presented as an exploratory descriptive index. Due to mathematical limitations (division by values near zero) and lack of validation, it should not be interpreted as a validated or robust indicator. These findings are hypothesis-generating and require confirmation in larger, more diverse populations. The findings also highlight the need for future studies with randomized or counterbalanced designs to rule out subtle order effects that may influence breath-hold performance and autonomic responses.

## 5. Limitations

Several limitations of this study should be considered when interpreting the results.

Confounding of respiratory phase and breath-hold duration. This is the most critical limitation of our study. Breath-hold duration was self-determined by each participant, resulting in considerable inter-individual variability. Crucially, expiration holds were systematically shorter than inspiration holds (median 32.1 s, IQR 25.4–39.0 s vs. median 56.3 s, IQR 48.4–65.8 s; difference ~24 s). Because phase and duration are almost perfectly collinear in our data (all inspiration holds were longer than all expiration holds), we cannot statistically separate their effects. The observed differences between inspiration and expiration holds may therefore reflect (i) true phase-dependent mechanisms (e.g., intrathoracic pressure, lung volume, mechanoreceptor engagement); (ii) duration-dependent mechanisms (e.g., differential chemoreflex activation, hypoxic/hypercapnic stress); or (iii) an interaction between both. Our exploratory correlation analysis within each phase was underpowered to resolve this question. We caution readers against interpreting our findings as evidence of phase-specific effects. All references to ‘inspiration-hold’ and ‘expiration-hold’ conditions in this manuscript should be understood as describing the experimental protocol, not as establishing causally distinct physiological states. Future studies must employ either (a) fixed-duration breath-holds across both phases or (b) a crossover design with matched apnea durations, to isolate the independent contribution of respiratory phase.

Inspiratory depth was not standardized. Participants were instructed to hold their breath at the end of inspiration or expiration, but the depth of the preceding inspiratory breath was not controlled. Deeper inspirations increase lung volume, reduce intrathoracic pressure more substantially, and may enhance venous return and stroke volume, thereby affecting baroreflex engagement. This uncontrolled variable may have contributed to inter-individual variability in ΔLFA responses.

*Fixed experimental order*. The experimental stages were performed in a fixed order rather than a randomized sequence. This design choice was made to ensure procedural consistency and minimize carryover effects between postures. However, it introduces the possibility that order effects, such as habituation, learning, or mild fatigue, could have influenced breath-hold durations and autonomic responses. Notably, expiration holds were consistently shorter than inspiration holds even though they appeared both early (Stage 2) and late (Stage 4) in the protocol, suggesting that the respiratory phase (and its associated mechanical conditions) is a strong determinant of breath-hold duration, though we cannot separate this from duration effects. Nevertheless, we cannot entirely exclude subtle order-dependent influences on autonomic parameters or breath-hold performance. Future studies employing randomized or counterbalanced designs would help to further disentangle these factors.

The episodic nature of the protocol, with alternating phases of breathing and breath-hold, resulted in short-duration segments for analysis. While time-domain methods and amplitude quantification are robust to this, it precluded the reliable calculation of very low-frequency components and detailed spectral decomposition requiring longer, stationary data.

While PPG is a valuable non-invasive tool for assessing vascular tone dynamics, it provides an indirect measure of peripheral resistance at a single site (typically the finger). It may not fully represent global systemic vascular resistance or central hemodynamic changes. The complexities of the PPG signal, which is influenced by a combination of neural, myogenic, endothelial, and hemodynamic factors, limit our ability to extrapolate our findings from the peripheral microcirculation to the systemic vasculature.

While we applied normalization to PPG signals to account for interindividual differences in signal gain, this approach has limitations. First, normalization to the mean value of the spontaneous breathing phase assumes that this baseline is stable and representative of the subject’s typical peripheral perfusion, which may not hold if baseline perfusion varies across stages or is influenced by the experimental protocol. Second, this procedure does not correct for differences in signal quality, noise characteristics, or motion artifacts that may affect LFA estimates. Future studies employing calibrated photoplethysmography or concurrent measurements of peripheral perfusion (e.g., laser Doppler flowmetry) could further improve interindividual comparability.

*Sample size and generalizability*. No formal a priori power calculation was performed. The sample size (n = 11) was pragmatic and exploratory. This study was adequately powered only to detect large effect sizes (Cohen’s d > 1.2) and is therefore strictly hypothesis-generating. While statistically significant differences were observed for certain comparisons (e.g., ΔLFA_PPG_ during expiration holds), the study was not adequately powered for robust subgroup analyses or to reliably detect small-to-moderate effect sizes. Therefore, non-significant findings should not be interpreted as evidence of no effect. Future studies with larger, more diverse samples and standardized breath-hold durations are warranted to confirm and extend these findings. Moreover, the study did not formally assess the effects of gender, baseline autonomic tone, or other individual characteristics on the observed responses. The sample size precludes meaningful subgroup analyses or multivariate modeling to adjust for these potential confounders.

*Wide confidence intervals and estimation uncertainty*. With 11 participants, bootstrap 95% confidence intervals around ΔLFA medians are very wide (e.g., Stage 2 ΔLFA_PPG_: 25.4% to 388.1%), indicating substantial individual variability and severe estimation uncertainty. These intervals should not be misinterpreted as precise population parameter estimates. Consequently, the reported *p*-values are sample-dependent, and false positive or false negative findings cannot be excluded. The small sample size precluded meaningful multivariate modeling that could account for potential confounders such as breath-hold duration, inspiratory depth, or baseline autonomic tone. Independent validation in a substantially larger sample is mandatory before any clinical or physiological generalization can be considered.

This study interprets changes in LF amplitude as indicative of altered autonomic outflows. However, LF oscillations in PPG are influenced by multiple mechanisms, including myogenic tone, endothelial function, and passive pressure–flow relationships. LF in HR variability reflects baroreflex gain rather than direct sympathetic cardiac tone. Without direct recordings of muscle sympathetic nerve activity (microneurography) or pharmacological blockade, our interpretations remain inferential. Readers should exercise caution when attributing observed changes to specific autonomic branches.

The heterogeneous responses observed, particularly the lack of significant correlations during longer inspiration-hold conditions, suggest that individual factors and testing conditions significantly influence the autonomic response to breath-hold. Future studies should explore the physiological determinants of these variable response patterns.

Division by ΔLFA_HR_ values that are near zero can produce unstable, arbitrarily large ratios. In our data, several ΔLFA_HR_ values were close to zero (e.g., Stage 3 median −13.27% with wide IQR), potentially amplifying ratio variability. We did not perform bootstrap resampling, outlier truncation, or sensitivity analyses for the ratio. Moreover, the physiological interpretation of the ratio is ambiguous: a negative ratio could arise from opposite directional changes (one signal increases, the other decreases) or from a small-magnitude denominator. Therefore, the ΔLFA ratio should be viewed as an exploratory descriptive tool only. Future studies should validate any ratio-based metric using larger samples, robust estimation methods, or alternative mathematical formulations.

The bootstrap confidence intervals for ΔLFA are wide, reflecting substantial interindividual variability and the exploratory nature of the study. These intervals should not be misinterpreted as precise estimates of population parameters.

Baroreflex engagement requires simultaneous recording of arterial blood pressure. Without blood pressure data, we cannot calculate baroreflex sensitivity, directly confirming or distinguishing between central and peripheral mechanisms. Consequently, all references to baroreflex in this manuscript should be understood as hypothesis-generating.

*Absence of respiratory gas and volume measurements*. This study recorded respiratory timing (nasal cannula flow signal) to identify breath-hold phases but did not measure tidal volume, end-tidal CO_2_ (EtCO_2_), or oxygen saturation (SpO_2_). Consequently, we cannot determine whether the observed autonomic responses were driven primarily by chemoreflex activation (hypoxia/hypercapnia), by mechanical effects (changes in intrathoracic pressure), or by central motor command. Inter-individual differences in baseline respiratory drive, inspiratory depth, or CO_2_ sensitivity may have contributed to the wide variability in ΔLFA responses. Without these measurements, our interpretations of chemoreflex or baroreflex mechanisms remain inferential. Future studies should incorporate capnography and pulse oximetry to quantify the physiological stimulus at the individual level.

Despite these limitations, the study provides valuable insights into the integrated cardiac and vascular autonomic response to provoked breath-hold, leveraging a multi-signal approach that enhances the traditional cardiocentric analysis.

## Figures and Tables

**Table 1:** Duration of breath-hold and spontaneous breathing phases across experimental stages.

*Stage No* *.*	*Condition*	*BH-Phase Duration, s*	*p-Level*	*SB-Phase Duration, s*
1	Supine, inspiration hold	53.5 (48.6, 62.2)	0.001	36.1 (35.2, 37.0)
2	Supine, expiration hold	34.0 (28.4, 38.9)		32.1 (30.4, 33.5)
3	Standing, inspiration hold	61.5 (48.9, 67.4)	0.002	33.8 (32.0, 34.4)
4	Standing, expiration hold	28.2 (25.4, 37.5)		30.6 (30.2, 31.4)
Total (1 + 3)	All inspiration holds	56.3 (48.4, 65.8)	<0.001	34.5 (33.5, 35.9)
Total (2 + 4)	All expiration holds	32.1 (25.4, 39.0)		30.9 (30.2, 33.4)
Total (All)	All stages	39.0 (30.8, 51.7)		32.2 (30.4, 34.6)

Data presented as median with lower and upper quartiles, Me (LQ, UQ). *p*-values were calculated using the Wilcoxon signed-rank test for paired samples (BH-phase duration: inspiration hold vs. expiration hold). BH-phase, breath-hold phase; SB-phase, spontaneous breathing phase.

**Table 2:** Parameters of HR variability, RSA and LF oscillations in HR and PPG across experimental stages.

*Parameter*	*Stage 1*	*Stage 2*	*Stage 3*	*Stage 4*	*p-Value*
HR variability:					
- SDNN_breathe_, ms	71.55 (61.73, 78.31)	37.03 (28.58, 48.57)	60.64 (34.97, 63.55)	29.77 (26.12, 42.18)	0.004
- SDNN_hold_, ms	54.78 (47.49, 74.56)	45.75 (31.82, 50.23)	59.95 (50.25, 75.42)	31.69 (28.92, 34.09)	0.172
- RMSSD_breathe_, ms	54.45 (41.07, 83.15)	33.07 (27.08, 47.05)	24.85 (19.50, 54.34)	22.99 (17.71, 28.31)	0.007
- RMSSD_hold_, ms	44.80 (27.76, 61.61)	29.22 (12.82, 36.16)	15.05 (12.91, 18.57)	14.24 (13.55, 17.95)	0.145
- SD1 _breathe_, ms	38.46 (29.02, 58.79)	23.38 (18.76, 33.26)	17.53 (13.78, 38.38)	16.25 (12.52, 20.01)	0.007
- SD1 _hold_, ms	31.37 (19.61, 43.56)	20.45 (8.83, 25.55)	10.54 (8.92, 12.92)	9.89 (9.30, 12.66)	0.145
- SD2 _breathe_, ms	80.09 (70.65, 96.15)	46.67 (30.44, 61.66)	67.58 (47.08, 81.51)	39.44 (32.23, 57.04)	0.005
- SD2 _hold_, ms	74.58 (59.75, 95.30)	45.12 (39.54, 63.49)	84.04 (68.12, 104.08)	43.50 (40.06, 47.08)	0.172
- Sample entropy _breathe_	1.45 (0.50, 2.15)	1.20 (0.28, 1.50)	1.32 (1.18, 1.90)	1.99 (1.39, 2.29)	0.033
- Sample entropy _hold_	0.75 (0.52, 1.21)	1.23 (0.78, 1.55)	0.41 (0.20, 0.72)	1.22 (0.89, 1.34)	0.284
RSA PtT _breathe_, ms	80.93 (50.75, 139.15)	71.75 (54.30, 106.76)	73.00 (54.13, 96.90)	48.89 (44.46, 61.45)	0.126
LFA in HR, ms:					
- LFA_breathe_	3.09 (2.37, 4.13)	1.92 (1.52, 3.30)	4.41 (2.50, 7.65)	2.18 (1.53, 3.11)	0.024
- LFA_hold_	1.54 (1.14, 3.05)	1.53 (1.14, 2.17)	2.72 (2.24, 3.10)	2.31 (2.18, 2.47)	0.122
LFA in PPG, a.u.:					
- LFA_breathe_	32.14 (27.32, 42.07)	23.03 (12.75, 41.48)	21.50 (11.52, 32.00)	21.54 (8.05, 54.81)	0.689
- LFA_hold_	37.84 (28.12, 57.40)	44.34 (30.04, 108.30)	23.25 (11.72, 28.10)	31.86 (20.34, 34.69)	0.060

Data presented as median with lower and upper quartiles, Me (LQ, UQ). *p*-values were calculated using the Friedman test for related samples and indicate the overall significance of differences across the four stages. Stage 1, supine inspiration hold; Stage 2, supine expiration hold; Stage 3, standing inspiration hold; Stage 4, standing expiration hold. HR, heart rate; PPG, photoplethysmogram; SDNN, standard deviation of RR intervals; RMSSD, root mean square of successive differences between RR intervals; SD1 and SD2, Poincaré plot parameters (SD1—short-term variability, SD2—long-term variability); RSA PtT, respiratory sinus arrhythmia peak-to-trough amplitude during spontaneous breathing phase; LFA, low-frequency oscillation amplitude; HR, heart rate; PPG, photoplethysmogram; a.u., arbitrary units; _breathe_, spontaneous breathing phase; _hold_, breath-hold phase.

**Table 3:** Relative change in LF oscillation amplitude from spontaneous breathing to breath-hold.

*Stage No* *.*	*Condition*	*ΔLFA_HR_, %*	*p-Value*	*ΔLFA_PPG_, %*	*p-Value*	*p-Value* (HR vs. PPG)	*Cliff’s Delta * (HR vs. PPG)	*ΔLFA Ratio*
1	Supine, inspiration hold	−52.42 (−60.41, 26.95)	0.383	18.46 (1.23, 55.71)	0.250	0.234	0.375	0.35 (−0.26, 0.55)
2	Supine, expiration hold	−32.38 (−42.22, −18.00)	0.020	71.56 (66.09, 122.61)	0.004	0.001	0.975	−2.21 (−4.85, −1.63)
3	Standing, inspiration hold	−13.27 (−62.00, 60.17)	0.844	−10.88 (−54.30, 35.25)	0.844	1.000	<0.001	0.80 (1.78, 0.23)
4	Standing, expiration hold	35.57 (−12.49, 127.54)	0.312	68.07 (20.97, 102.69)	0.219	1.000	<0.001	0.73 (0.70, 0.97)
Total (1 + 3)	All inspiration holds	−41.39 (−63.58, 42.41)	0.715	10.03 (−16.07, 46.99)	0.502	0.370	0.245	−0.14 (−0.86, 0.36)
Total (2 + 4)	All expiration holds	−19.84 (−37.70, 17.10)	0.524	71.56 (44.62, 113.35)	0.003	0.005	0.600	−1.63 (−3.22, 0.73)
Total (All)	All stages	−25.28 (−52.59, 24.64)	0.481	49.75 (6.67, 106.65)	0.003	0.012		−0.25 (−2.07, 0.70)

Data presented as median with lower and upper quartiles, Me (LQ, UQ). *p*-values were calculated using the Wilcoxon signed-rank test and indicate the significance of the change in LFA within each stage, i.e., whether the ΔLFA is significantly different from zero. *p*-values (HR vs. PPG) were calculated using the Mann–Whitney U test and indicate the significance of the difference in the relative change (ΔLFA, %) between the HR and PPG signals within the same stage. ΔLFA_HR_ and ΔLFA_PPG_, relative change in LF oscillation amplitude in HR and PPG signals from spontaneous breathing to breath-hold, respectively. ΔLFA ratio, the ratio of ΔLFA_PPG_ to ΔLFA_HR_. HR, heart rate; PPG, photoplethysmogram; LFA, low-frequency oscillation amplitude. Stage 1: Bootstrap 95% CI for the median ΔLFA_HR_, [−65.2, 50.1]; for the median ΔLFA_PPG_, [−16.9, 106.6]. Stage 2: Bootstrap 95% CI for the median ΔLFA_HR_, [−58.7, −7.3]; for the median ΔLFA_PPG_, [25.4, 388.1]. Stage 3: Bootstrap 95% CI for the median ΔLFA_HR_, [−74.8, 125.1]; for the median ΔLFA_PPG_, [−70.3, 104.8]. Stage 4: Bootstrap 95% CI for the median ΔLFA_HR_, [−27.4, 155.2]; for the median ΔLFA_PPG_, [−35.3, 113.4]. All inspiration holds: Bootstrap 95% CI for the median ΔLFA_HR_, [−65.2, 50.1]; for the median ΔLFA_PPG_, [−16.9, 49.8]. All expiration holds: Bootstrap 95% CI for the median ΔLFA_HR_, [−40.4, 9.6]; for the median ΔLFA_PPG_, [63.9, 113.9].

## Data Availability

The parameters presented in this study are available on reasonable request from the corresponding author. The primary data are not publicly available due to the policy of access to clinical data of the Saratov State Medical University.
